# Farmers' Funds for Hospitals

**Published:** 1921-03-05

**Authors:** 


### Farmers' Funds for Hospitals.

### THE NEW LANCASTER SCHEME.

The Preston and County of Lancaster Royal Infirmary has recently received a handsome cheque for £2,247 10s. from agricultural friends in West Lancashire. The effort was the outcome of a suggestion made to the farmers attending the Preston Cattle Market by Mr. John Gibson, Superintendent and Secretary of the hospital. On "Farmers' Day" gifts of produce and live stock were sold by auction, and, in addition, house-to-house collections were made in over seventy townships.

Mr. E. G. Hothersall, J.P., Chairman of the Fund, when handing over the cheque, said that the widespread expressions of gratitude for the institution's work and the general willingness to subscribe had made the duties of himself and his friends a real pleasure. As a token of appreciation of the efforts of all, the Board presented Mr. Hothersall with a silver salver, suitably inscribed.

The finances of the hospital are in a very satisfactory condition. During the last three years a debt of £2,200 has been paid off, the income increased from £8,900 to £21,300, and £9,000 raised for extensions (without a general appeal and exclusive of legacies); a new orthopædic department, a venereal disease centre, and a new ward of twenty beds are being built, and an auxiliary hospital of fifty beds at Lostock Hall is to be opened before the summer.

The workpeople's collections are being reorganised on the basis of 2d. per week per employee. Mr. Gibson has for some time been engaged on propaganda, which is showing very gratifying results.

